# Supplementary material for: Study on the Relationships between Doctor Characteristics and Online Consultation Volume in the Online Medical Community
Source: Healthcare (Basel). 2022 Aug 16;10(8):1551. doi: 10.3390/healthcare10081551 (PMC9408720; doi:10.3390/healthcare10081551)
Supplement: Supplementary file 1 [file healthcare-10-01551-s001.zip › healthcare-1828772-supplementary.pdf]

Table S1. Comments Comparison

Number	Program	Artificiality	Match
		Leukemia	
1	Pos	Pos	Yes
2	Pos	Pos	Yes
3	Pos	Pos	Yes
4	Pos	Pos	Yes
5	Pos	Pos	Yes
6	Pos	Pos	Yes
7	Pos	Pos	Yes
8	Pos	Pos	Yes
9	Pos	Pos	Yes
10	Pos	Pos	Yes
11	Pos	Pos	Yes
12	Neg	Neu	No
13	Pos	Pos	Yes
		Diabetes	
1	Pos	Pos	Yes
2	Pos	Pos	Yes
3	Pos	Pos	Yes
4	Neu	Pos	No
5	Pos	Pos	Yes
6	Pos	Pos	Yes
7	Pos	Pos	Yes
8	Neg	Neg	Yes
9	Pos	Pos	Yes
10	Pos	Pos	Yes
11	Pos	Pos	Yes
12	Pos	Pos	Yes
13	Pos	Pos	Yes
14	Neg	Neg	Yes
15	Pos	Pos	Yes
16	Pos	Pos	Yes
17	Pos	Pos	Yes
18	Neg	Pos	No
19	Pos	Pos	Yes
20	Pos	Pos	Yes
21	Pos	Pos	Yes
22	Pos	Pos	Yes
23	Pos	Pos	Yes
24	Pos	Pos	Yes
25	Neu	Pos	No
26	Pos	Pos	Yes
27	Pos	Pos	Yes
28	Neu	Pos	No
29	Pos	Neu	No
30	Pos	Pos	Yes
31	Pos	Pos	Yes
32	Pos	Pos	Yes
33	Pos	Pos	Yes

34	Neu	Pos	No
35	Pos	Pos	Yes
36	Pos	Pos	Yes
37	Pos	Pos	Yes
38	Pos	Pos	Yes
39	Pos	Pos	Yes
Depression			
1	Neg	Neg	Yes
2	Pos	Pos	Yes
3	Pos	Pos	Yes
4	Pos	Pos	Yes
5	Pos	Pos	Yes
6	Pos	Pos	Yes
7	Neu	Pos	No
8	Pos	Pos	Yes
9	Neu	Pos	No
10	Pos	Pos	Yes
11	Pos	Pos	Yes
12	Neg	Neu	No
13	Pos	Pos	Yes
14	Pos	Pos	Yes
15	Pos	Pos	Yes
16	Pos	Pos	Yes
17	Pos	Pos	Yes
18	Pos	Pos	Yes
19	Pos	Pos	Yes
20	Pos	Pos	Yes
21	Pos	Pos	Yes
22	Pos	Pos	Yes
23	Neu	Neu	Yes
24	Pos	Pos	Yes
25	Pos	Pos	Yes
26	Neg	Pos	No
27	Pos	Pos	Yes
28	Pos	Pos	Yes
29	Pos	Pos	Yes
30	Pos	Pos	Yes
31	Neg	Neu	No
32	Pos	Pos	Yes
33	Pos	Pos	Yes
34	Pos	Pos	Yes
35	Pos	Pos	Yes
36	Pos	Pos	Yes
37	Pos	Pos	Yes
38	Pos	Pos	Yes
39	Pos	Pos	Yes
40	Pos	Pos	Yes
41	Pos	Pos	Yes
42	Pos	Pos	Yes
43	Pos	Pos	Yes
44	Pos	Pos	Yes

45	Neu	Pos	No
46	Pos	Pos	Yes
47	Pos	Pos	Yes
48	Pos	Pos	Yes
Total	100	Matching Rate	87%
